# Crystal structure of 4′-{[4-(2,2′:6′,2′′-terpyrid­yl-4′-yl)phen­yl]ethyn­yl}biphenyl-4-yl (2,2,5,5-tetra­methyl-1-oxyl-3-pyrrolin-3-yl)formate benzene 2.5-solvate

**DOI:** 10.1107/S2056989015017697

**Published:** 2015-09-26

**Authors:** Andreas Meyer, Gregor Schnakenburg, Olav Schiemann

**Affiliations:** a University of Bonn, Institute of Physical and Theoretical Chemistry, Wegelerstr. 12, 53115 Bonn, Germany; b University of Bonn, Institute of Inorganic Chemistry, Gerhard-Domagk-Strasse 1, 53121 Bonn, Germany

**Keywords:** crystal structure, terpyridine, nitrox­yl, nitroxide, phenyl­ethynylbiphen­yl, ethynylphen­yl, C—H⋯π inter­actions, π–π inter­actions, hydrogen bonds

## Abstract

The title compound, C_44_H_35_N_4_O_3_·2.5C_6_H_6_ (**1**), consists of a terpyridine and a *N*-oxylpyrroline-3-formate group separated by an aromatic spacer, *viz.* 4-(phenyl­ethyn­yl)-1,1′-biphenyl. It crystallized in the triclinic space group *P*-1 with two and a half benzene solvate mol­ecules (one benzene mol­ecule is located about an inversion center), while the di­chloro­methane solvate (**2**) of the same mol­ecule [Ackermann *et al.* (2015 ▸). *Chem. Commun.* **51**, 5257–5260] crystallized in the tetra­gonal space group *P*4_2_/*n*, with considerable disorder in the mol­ecule. In (**1**), the terpyridine (terpy) group assumes an all-*trans* conformation typical for terpyridines. It is essentially planar with the two outer pyridine rings (*B* and *C*) inclined to the central pyridine ring (*A*) by 8.70 (15) and 14.55 (14)°, respectively. The planes of the aromatic spacer (*D*, *E* and *F*) are nearly coplanar with dihedral angles *D*/*E*, *D*/*F* and *E*/*F* being 3.42 (15), 5.80 (15) and 4.00 (16)°, respectively. It is twisted with respect to the terpy group with, for example, dihedral angle *A*/*D* being 24.48 (14)°. The mean plane of the *N*-oxylpyrroline is almost normal to the biphenyl ring *F*, making a dihedral angle of 86.57 (16)°, and it is inclined to pyridine ring *A* by 72.61 (15)°. The intra­molecular separation between the O atom of the nitroxyl group and the N atom of the central pyridine ring of the terpyridine group is 25.044 (3) Å. In the crystal, mol­ecules are linked by pairs of C—H⋯O hydrogen bonds, forming inversion dimers. The dimers stack along the *c* axis forming columns. Within and between the columns, the spaces are occupied by benzene mol­ecules. The shortest oxygen–oxygen separation between nitroxyl groups is 4.004 (4) Å. The details of the title compound are compared with those of the di­chloro­methane solvate (**2**) and with the structure of a related mol­ecule, 4′-{4-[(2,2,5,5-tetra­methyl-*N*-oxyl-3-pyrrolin-3-yl)ethyn­yl]phen­yl}-2,2′:6′,2′′-terpyridine (**3**), which has an ethynylphenyl spacer [Meyer *et al.* (2015). *Acta Cryst.* E**71**, 870–874].

## Abstract 2

The title compound, consisting of a terpyridine group linked to a *N*-oxylpyrroline-3-formate group by a phenyl­ethynylbiphenyl spacer, crystallized as a benzene two and a half solvate. Its structure is compared to that of the same mol­ecule that crystallized as a di­chloro­methane solvate and to a similar mol­ecule with a shorter spacer unit *viz.* ethynylphenyl.

## Chemical context   

The title compound (**1**) was synthesized as a ligand for 3*d* metal ions in the framework of a pulsed EPR study on metal–nitroxyl model systems. It contains a nitroxyl group and a terpyridine (terpy) group which is capable of taking up metal ions. The title compound resembles compound (**3**) (4′-{4-[(2,2,5,5-tetra­methyl-*N*-oxyl-3-pyrrolin-3-yl)ethyn­yl]phen­yl}-2,2′:6′,2′′-terpyridine), which has an ethynylphenyl spacer (Meyer *et al.*, 2015*a* ▸), compared to the phenyl­ethynylbiphenyl spacer in the title compound (**1**). Nitroxyls are of inter­est in various branches of chemistry including magnetochemistry (Rajca *et al.*, 2006 ▸; Fritscher *et al.*, 2002 ▸), synthetic chemistry (Hoover & Stahl, 2011 ▸; Fey *et al.*, 2001 ▸) and structural biology (Reginsson & Schiemann, 2011 ▸). Terpyridines show pH-dependent luminescence properties which have been analyzed in terms of a pH-dependent *cis*–*trans* isomerization (Nakamoto, 1960 ▸; Fink & Ohnesorge, 1970 ▸). Structural investigations in the solid state reveal an exclusive preference for the *trans* conformation (Fallahpour *et al.*, 1999 ▸; Eryazici *et al.*, 2006 ▸; Bessel *et al.*, 1992 ▸; Grave *et al.*, 2003 ▸). Terpyridines have been shown to be versatile ligands for various metal ions (Hogg & Wilkins, 1962 ▸; Constable *et al.*, 1999 ▸; Narr *et al.*, 2002 ▸; Meyer *et al.*, 2015*b* ▸; Folgado *et al.*, 1990 ▸).
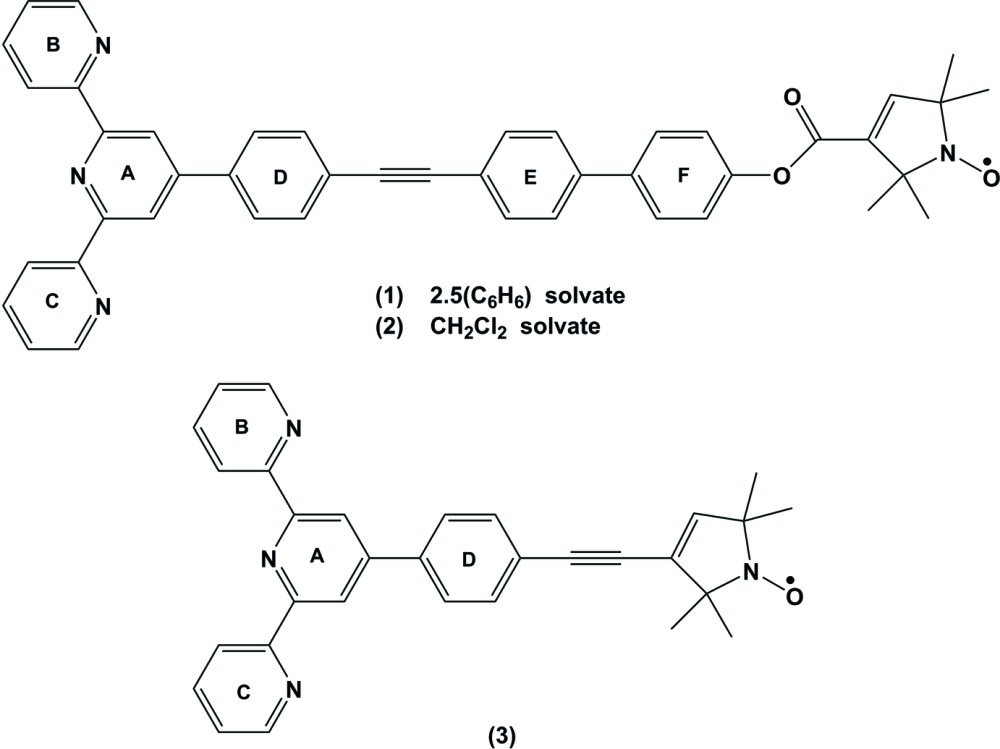


## Structural commentary   

**Figure 1:**
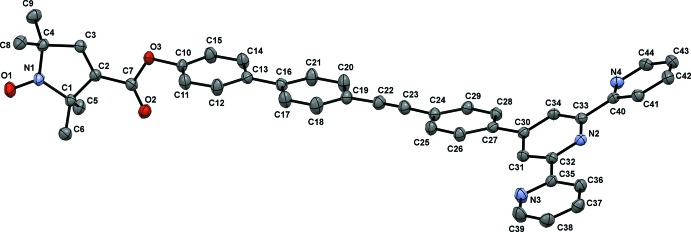
The mol­ecular structure of the title compound (**1**), with atom labelling. Displacement ellipsoids are drawn at 50% probability level. The benzene mol­ecules and the H atoms have been omitted for clarity.

**Figure 2:**
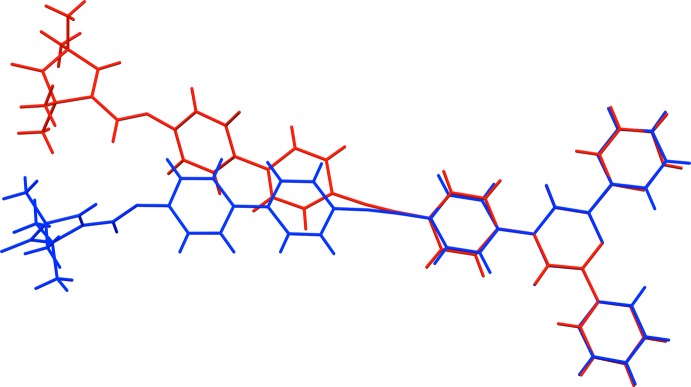
The structural overlay of compounds (**1**) and (**2**) [title compound (**1**) blue, compound (**2** – the di­chloro­methane solvate (Ackermann *et al.*, 2015 ▸) – red].

**Figure 3:**
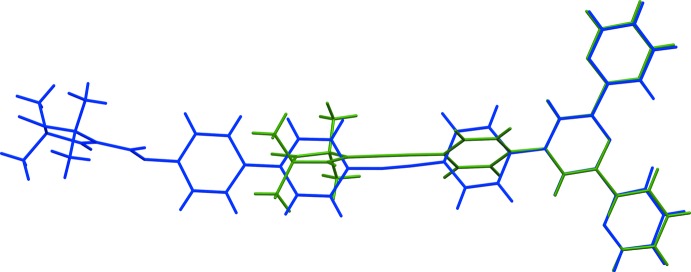
The structural overlay of compounds (**1**) and (**3**) [title compound (**1**) blue, compound (**3**) – (Meyer *et al.*, 2015*a* ▸) – green].

The mol­ecular structure of the title compound, (**1**), is shown in Fig. 1 ▸. The crystal structure of the di­chloro­methane solvate (**2**) of the title compound has been reported (Ackermann *et al.*, 2015 ▸). However, these authors used a different protocol for the crystallization of (**1**) and the conformation of (**2**) differs markedly from the one presented herein, as shown in the structural overlay of the two compounds (Fig. 2 ▸). The structural overlay of compounds (**1**) and (**3**) also illustrate the differences in their conformations (Fig. 3 ▸).

In (**1**) the terpy group assumes the usual all–*trans* conformation (Meyer *et al.*, 2015*a* ▸; Fallahpour *et al.*, 1999 ▸; Eryazici *et al.*, 2006 ▸; Bessel *et al.*, 1992 ▸; Grave *et al.*, 2003 ▸). It is essentially planar with the two outer rings *B* (N3/C35–C39) and *C* (N4/C40–C44) being inclined to the central pyridine ring *A* (N2/C30–C34) by 8.70 (15) and 14.55 (14)°, respectively. The conformation of the nitroxyl group in (1) is similar to that found in (**3**), with a planar pyrroline (N1/C1–C4) ring assuming an angle of 72.61 (15)° to the central pyridine ring *A* [see also Margraf *et al.* (2009 ▸) and Schuetz *et al.* (2010 ▸)]. In (**3**) this dihedral angle is 88.44 (7)°, while in (**2**) the same dihedral angle is 21.6 (2)°.

The *N*-oxylpyrroline-3-formate subunit is linked by a rigid spacer, consisting of a 4,4′-bi­phenyl­ene, an ethynylene and a *p*-phenyl­ene group, to the terpy subunit. The intra­molecular separation of the nitroxyl and the terpy group is 25.044 (3) Å (measured between O1 and N2). The three phenyl groups within the spacer are nearly coplanar, with dihedral angles between the rings of 4.00 (16)°, for rings *D* (C10–C15) and *E* (C16–C21), and 3.42 (15)° for rings *E* and *F* (C24–C29). Compared to the structure of (**3**), the spacer is closer to coplanarity to the central pyridine ring: dihedral angle *A*/*D* is 24.48 (14)°, compared to 51.36 (7)° in (**3**). The ethynylene group is slightly bent as in (**3**), with angle C19–C22–C23 = 174.6 (3) and C22–C23–C24 = 177.8 (3)°. There are short C—H⋯N contacts in the mol­ecule of 2.48 Å (H31⋯N3) and 2.49 Å (H34⋯N4). The same short contacts are also observed in (**3**). Such contacts have been classified as hydrogen bonds by Murguly *et al.* (1999 ▸).

## Supra­molecular features   

**Figure 4:**
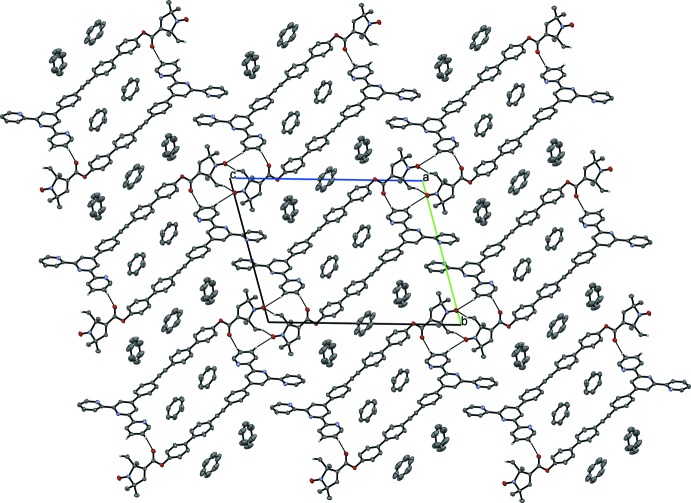
Crystal packing of the title compound viewed along the *a* axis. Weak C—H⋯O hydrogen bonds are shown as dashed lines (see Table 1 ▸). H atoms not involved in C—H⋯O bonds have been omitted for clarity.

**Figure 5:**
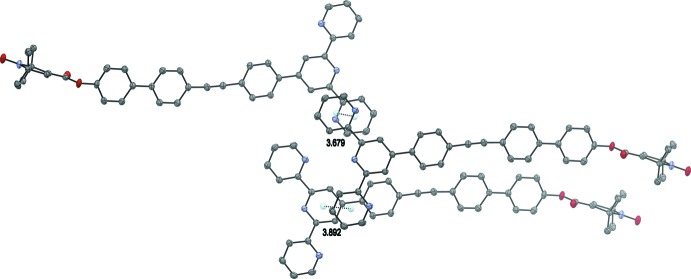
π-stacking inter­actions between pyridine rings of neighboring mol­ecules. H atoms have been omitted for clarity.

In the crystal of (**1**), Fig. 4 ▸, mol­ecules form layers which are nearly coplanar with the (0
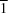
1) plane. Neighbouring layers differ in the orientation of the mol­ecules and each layer is separated by layers of solvent mol­ecules. This arrangement possibly leads to favorable dispersive inter­actions although only one short C—H⋯π contact is observed between the solvent mol­ecules and mol­ecules of (**1**) (Table 1 ▸). Short π–π contacts are observed between the *C* rings of neighbouring mol­ecules and between the *B* and *C* rings (Fig. 5 ▸). The centroid-to-centroid distances are 3.678 (2) and 3.8915 (18) Å, respectively, and can be classified as slipped face-to-face π-inter­actions (Janiak, 2000 ▸).

Within the planes, there are weak C—H⋯O hydrogen bonds between the nitroxyl-O atom and the *para-*hydrogen atom of pyridine ring *B* (Table 1 ▸). Furthermore, two weak hydrogen bonds per mol­ecule are formed between pairs of layers (Table 1 ▸). One of these hydrogen bonds involves the nitroxyl O atom and a hydrogen atom of a methyl group of a mol­ecule from a neighboring layer. The other hydrogen bond is formed between the carbonylic O atom of the carboxyl­ate group and a *meta*-hydrogen atom of one of the outer pyridine rings of a mol­ecule from a neighboring layer. As the layers are hydrogen bonded pair-wise, the structure can also be described as consisting of double-layers.

It is noteworthy that the arrangement of the mol­ecules of the title compound strongly depends upon the solvents of crystallization. In compound (**1**), the mol­ecules are arranged in layers and the benzene mol­ecules fill out the channels between the layers formed by the aromatic spacers of the mol­ecule. Close inter­molecule contacts exist only between the functional groups. In the structure of (**2**) (Ackermann *et al.*, 2015 ▸), the solvent of crystallization is di­chloro­methane instead of benzene and mol­ecules are arranged having fourfold rotational site symmetry. The solvent mol­ecules fill out channels between the mol­ecules of (**2**), as in (**1**). However, the CH_2_Cl_2_ solvent mol­ecules in (**2**) are in close proximity to the terpyridine groups instead of to the aromatic spacer. Weak hydrogen bonds are formed predominantly involving the O atoms as acceptors and the pyrroline and the pyridine rings as donors, as observed in (**2**) and (**3**). The shortest oxygen–oxygen separation between neighboring nitroxyl groups is 4.004 (4) Å. This O⋯O distance is an important factor determining the strength of through space exchange inter­actions of nitroxyls (Rajca *et al.* 2006 ▸).

## Database survey   

The Cambridge Structural Database (CSD, Version 5.36; Groom & Allen, 2014 ▸) has not been updated since our presentation of the structure of (**2**). The CSD query revealed, that non-coordinated terpyridines are arranged in an all-*trans* conformation, unless they are either protonated, li­thia­ted or cannot assume an all-*trans* conformation for reasons of steric hindrance.

## Synthesis and crystallization   

**Figure 6:**
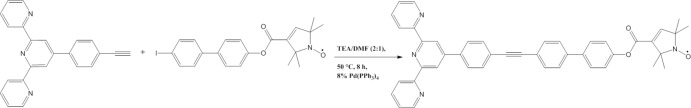
The synthesis of the title compound (**1**).

The synthesis of the title compound (**1**), is illustrated in Fig. 6 ▸. 480 mg (1.45 mmol) of 4′-(4-ethynylphen­yl)-2,2′:6′,2′′-terpyridine (Grosshenny & Ziessel, 1993 ▸), 780 mg (1.69 mmol) of 4′-iodo-*p*-biphen-4-yl-*N*-oxyl-2,2,5,5-tetra­methyl­pyrroline-3-formate (Bode *et al.*, 2008 ▸) and 85 mg (0.12 mmol) of tetra­kis­(tri­phenyl­phosphane)palladium(0) were dissolved in a mixture of 20 ml of tri­ethyl­amine (TEA) and 9 ml of di­methyl­formamide (DMF) giving rise to an orange solution. The solution was heated to 323 K and stirred for 8 h after which the solvents were removed under reduced pressure. The resulting dark-orange powder was dissolved in di­chloro­methane (DCM) and subjected to column chromatography using aluminum oxide (5% water, height 30 cm, diameter 2.3 cm). First, a mixture of DCM and hexane in a 1:2 ratio was used as eluent until all remaining educt, reagents and side products were eluted (approximately 200–300 ml). The column was then eluted using pure DCM to obtain a yellow solution. Removing the solvent yielded the product as a pale-yellow solid (yield: 90%). Crystals suitable for X-ray crystallography were obtained by layering a solution of (**1**) in benzene with *n*-hexane.

## Refinement   

Crystal data, data collection and structure refinement details are summarized in Table 2 ▸. The H atoms were included in calculated positions and treated as riding atoms: C-H = 0.95-0.98 Å with U_iso_(H) = 1.5U_eq_(C) for methyl H atoms and 1.2U_eq_(C) for other H atoms. 16 reflections with bad agreement were omitted from the final refinement cycles.

## Supplementary Material

Crystal structure: contains datablock(s) I. DOI: 10.1107/S2056989015017697/su5206sup1.cif

Structure factors: contains datablock(s) I. DOI: 10.1107/S2056989015017697/su5206Isup2.hkl

CCDC reference: 1426093

Additional supporting information:  crystallographic information; 3D view; checkCIF report

## Figures and Tables

**Table 1:** Hydrogen-bond geometry (, ) *Cg*4, *Cg*7 and *Cg*10 are the centroids of pyridine ring N4/C40C44, spacer ring C24C29 and benzene ring C54C59, respectively.

*D*H*A*	*D*H	H*A*	*D* *A*	*D*H*A*
C37H37O1^i^	0.95	2.65	3.228(4)	120
C38H38O2^ii^	0.95	2.55	3.485(4)	169
C6H6*C*O1^iii^	0.98	2.61	3.499(4)	151
C9H9*B* *Cg*4^iv^	0.96	2.79	3.602(4)	140
C14H14*Cg*10^v^	0.95	2.88	3.608(4)	134
C14H14*Cg*10^vi^	0.95	2.88	3.608(4)	134
C55H55*Cg*7^vii^	0.95	2.90	3.680(3)	140

Symmetry codes: (i) 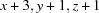; (ii) 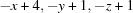; (iii) 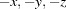; (iv) 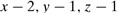; (v) 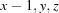; (vi) 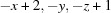; (vii) 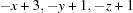.

**Table 2:** Experimental details

Crystal data	
Chemical formula	C_44_H_35_N_4_O_3_2.5C_6_H_6_
*M*_r_	863.03
Crystal system, space group	Triclinic, *P* 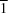
Temperature (K)	123
*a*, *b*, *c* ()	5.7578(1), 18.0559(4), 23.3716(6)
, , ()	105.5870(13), 93.7408(13), 92.6002(14)
*V* (^3^)	2330.41(9)
*Z*	2
Radiation type	Mo *K*
(mm^1^)	0.08
Crystal size (mm)	0.28 0.20 0.08

Data collection	
Diffractometer	Nonius KappaCCD
Absorption correction	Multi-scan (*SORTAV*; Blessing, 1995 ▸)
*T*_min_, *T*_max_	0.808, 1.000
No. of measured, independent and observed [*I* > 2(*I*)] reflections	74528, 11227, 6356
*R*_int_	0.109
(sin /)_max_ (^1^)	0.661

Refinement	
*R*[*F* ^2^ > 2(*F* ^2^)], *wR*(*F* ^2^), *S*	0.071, 0.217, 1.07
No. of reflections	11227
No. of parameters	587
No. of restraints	1
H-atom treatment	H-atom parameters constrained
_max_, _min_ (e ^3^)	0.33, 0.27

Computer programs: *HKL* *DENZO* and *SCALEPACK* (Otwinowski Minor 1997 ▸), *SHELXS97* (Sheldrick,2008 ▸), *SHELXL2013* (Sheldrick, 2015 ▸), *OLEX2* (Dolomanov *et al.*, 2009 ▸) and *Mercury* (Macrae *et al.*, 2008 ▸).

## supplementary crystallographic information

### Crystal data

**Table:**

C_44_H_35_N_4_O_3_·2.5C_6_H_6_	*Z* = 2
*M**_r_* = 863.03	*F*(000) = 912
Triclinic, *P*1	*D*_x_ = 1.230 Mg m^−^^3^
*a* = 5.7578 (1) Å	Mo *K*α radiation, λ = 0.71073 Å
*b* = 18.0559 (4) Å	Cell parameters from 12020 reflections
*c* = 23.3716 (6) Å	θ = 1.0–29.1°
α = 105.5870 (13)°	µ = 0.08 mm^−^^1^
β = 93.7408 (13)°	*T* = 123 K
γ = 92.6002 (14)°	Plate, yellow
*V* = 2330.41 (9) Å^3^	0.28 × 0.20 × 0.08 mm

### Data collection

**Table:**

Nonius KappaCCD diffractometer	6356 reflections with *I* > 2σ(*I*)
fine slicing φ and ω scans	*R*_int_ = 0.109
Absorption correction: multi-scan (*SORTAV*; Blessing, 1995)	θ_max_ = 28.0°, θ_min_ = 1.8°
*T*_min_ = 0.808, *T*_max_ = 1.000	*h* = −7→7
74528 measured reflections	*k* = −23→23
11227 independent reflections	*l* = −30→30

### Refinement

**Table:**

Refinement on *F*^2^	Primary atom site location: structure-invariant direct methods
Least-squares matrix: full	Secondary atom site location: difference Fourier map
*R*[*F*^2^ > 2σ(*F*^2^)] = 0.071	Hydrogen site location: inferred from neighbouring sites
*wR*(*F*^2^) = 0.217	H-atom parameters constrained
*S* = 1.07	*w* = 1/[σ^2^(*F*_o_^2^) + (0.0715*P*)^2^ + 2.806*P*] where *P* = (*F*_o_^2^ + 2*F*_c_^2^)/3
11227 reflections	(Δ/σ)_max_ < 0.001
587 parameters	Δρ_max_ = 0.33 e Å^−^^3^
1 restraint	Δρ_min_ = −0.27 e Å^−^^3^

### Special details

**Table:**

Geometry. All e.s.d.'s (except the e.s.d. in the dihedral angle between two l.s. planes) are estimated using the full covariance matrix. The cell e.s.d.'s are taken into account individually in the estimation of e.s.d.'s in distances, angles and torsion angles; correlations between e.s.d.'s in cell parameters are only used when they are defined by crystal symmetry. An approximate (isotropic) treatment of cell e.s.d.'s is used for estimating e.s.d.'s involving l.s. planes.

### Fractional atomic coordinates and isotropic or equivalent isotropic displacement parameters (Å^2^)

**Table:**

	*x*	*y*	*z*	*U*_iso_*/*U*_eq_	
C1	0.2370 (5)	−0.01295 (16)	0.10190 (12)	0.0227 (6)	
C2	0.3869 (5)	−0.03265 (16)	0.15104 (12)	0.0228 (6)	
C3	0.4509 (5)	−0.10465 (16)	0.13551 (13)	0.0243 (6)	
H3	0.5452	−0.1260	0.1611	0.029*	
C4	0.3597 (5)	−0.14879 (16)	0.07364 (13)	0.0258 (6)	
C5	−0.0104 (5)	0.00458 (18)	0.11846 (14)	0.0299 (7)	
H5A	−0.1095	0.0036	0.0825	0.045*	
H5B	−0.0082	0.0557	0.1468	0.045*	
H5C	−0.0727	−0.0343	0.1367	0.045*	
C6	0.3447 (5)	0.04980 (17)	0.07767 (14)	0.0294 (7)	
H6A	0.5016	0.0368	0.0661	0.044*	
H6B	0.3544	0.0991	0.1085	0.044*	
H6C	0.2475	0.0539	0.0428	0.044*	
C7	0.4547 (5)	0.02506 (17)	0.20817 (13)	0.0242 (6)	
C8	0.5527 (5)	−0.17353 (18)	0.03214 (14)	0.0319 (7)	
H8A	0.4832	−0.1971	−0.0085	0.048*	
H8B	0.6441	−0.2110	0.0456	0.048*	
H8C	0.6548	−0.1284	0.0326	0.048*	
C9	0.1932 (5)	−0.21795 (18)	0.07291 (15)	0.0343 (7)	
H9A	0.0694	−0.2006	0.0993	0.051*	
H9B	0.2799	−0.2562	0.0867	0.051*	
H9C	0.1238	−0.2412	0.0322	0.051*	
C10	0.6996 (5)	0.04666 (16)	0.29676 (13)	0.0280 (7)	
C11	0.9052 (6)	0.08515 (19)	0.29138 (14)	0.0358 (7)	
H11	0.9637	0.0790	0.2534	0.043*	
C12	1.0255 (6)	0.13289 (19)	0.34188 (14)	0.0342 (7)	
H12	1.1682	0.1591	0.3381	0.041*	
C13	0.9437 (5)	0.14379 (16)	0.39818 (13)	0.0256 (6)	
C14	0.7358 (5)	0.10257 (19)	0.40168 (14)	0.0330 (7)	
H14	0.6767	0.1076	0.4395	0.040*	
C15	0.6137 (6)	0.05439 (19)	0.35106 (14)	0.0342 (7)	
H15	0.4721	0.0271	0.3542	0.041*	
C16	1.0710 (5)	0.19732 (17)	0.45189 (13)	0.0253 (6)	
C17	1.2858 (6)	0.23439 (19)	0.44816 (14)	0.0360 (8)	
H17	1.3476	0.2258	0.4104	0.043*	
C18	1.4098 (6)	0.2824 (2)	0.49680 (14)	0.0361 (8)	
H18	1.5546	0.3067	0.4922	0.043*	
C19	1.3264 (5)	0.29639 (17)	0.55323 (13)	0.0285 (7)	
C20	1.1098 (6)	0.2615 (2)	0.55805 (14)	0.0366 (8)	
H20	1.0467	0.2711	0.5957	0.044*	
C21	0.9861 (5)	0.21278 (19)	0.50809 (14)	0.0345 (7)	
H21	0.8395	0.1893	0.5123	0.041*	
C22	1.4677 (5)	0.34418 (17)	0.60378 (14)	0.0299 (7)	
C23	1.6001 (5)	0.38338 (17)	0.64283 (13)	0.0288 (7)	
C24	1.7619 (5)	0.43207 (17)	0.68813 (13)	0.0273 (6)	
C25	1.9752 (5)	0.45919 (17)	0.67281 (13)	0.0296 (7)	
H25	2.0113	0.4455	0.6324	0.036*	
C26	2.1331 (5)	0.50571 (17)	0.71624 (13)	0.0285 (7)	
H26	2.2755	0.5242	0.7051	0.034*	
C27	2.0868 (5)	0.52596 (15)	0.77632 (13)	0.0235 (6)	
C28	1.8728 (5)	0.49925 (16)	0.79124 (13)	0.0256 (6)	
H28	1.8368	0.5129	0.8317	0.031*	
C29	1.7132 (5)	0.45334 (16)	0.74799 (13)	0.0265 (6)	
H29	1.5689	0.4361	0.7591	0.032*	
C30	2.2616 (5)	0.57301 (15)	0.82326 (12)	0.0230 (6)	
C31	2.4348 (5)	0.62120 (16)	0.80997 (13)	0.0247 (6)	
H31	2.4364	0.6278	0.7710	0.030*	
C32	2.6059 (5)	0.65965 (16)	0.85480 (12)	0.0241 (6)	
C33	2.4383 (5)	0.60956 (16)	0.92376 (13)	0.0240 (6)	
C34	2.2643 (5)	0.56824 (16)	0.88191 (12)	0.0244 (6)	
H34	2.1482	0.5370	0.8931	0.029*	
C35	2.7986 (5)	0.70819 (16)	0.84142 (13)	0.0249 (6)	
C36	2.9585 (5)	0.75177 (17)	0.88581 (14)	0.0292 (7)	
H36	2.9416	0.7538	0.9264	0.035*	
C37	3.1439 (5)	0.79252 (17)	0.87052 (14)	0.0310 (7)	
H37	3.2553	0.8228	0.9004	0.037*	
C38	3.1634 (6)	0.78823 (18)	0.81163 (14)	0.0337 (7)	
H38	3.2898	0.8146	0.7996	0.040*	
C39	2.9941 (6)	0.7444 (2)	0.77017 (15)	0.0388 (8)	
H39	3.0071	0.7421	0.7294	0.047*	
C40	2.4478 (5)	0.60351 (15)	0.98644 (12)	0.0236 (6)	
C41	2.6483 (5)	0.62869 (17)	1.02509 (13)	0.0279 (6)	
H41	2.7844	0.6482	1.0117	0.033*	
C42	2.6446 (6)	0.62468 (18)	1.08338 (13)	0.0320 (7)	
H42	2.7765	0.6429	1.1111	0.038*	
C43	2.4456 (6)	0.59362 (18)	1.10055 (14)	0.0333 (7)	
H43	2.4384	0.5898	1.1402	0.040*	
C44	2.2573 (6)	0.56820 (18)	1.05891 (14)	0.0324 (7)	
H44	2.1224	0.5461	1.0709	0.039*	
C45	0.4296 (6)	0.3900 (2)	0.35899 (16)	0.0421 (8)	
H45	0.2895	0.3889	0.3349	0.050*	
C46	0.4922 (7)	0.4522 (2)	0.40689 (16)	0.0465 (9)	
H46	0.3934	0.4938	0.4163	0.056*	
C47	0.6977 (7)	0.4549 (2)	0.44152 (17)	0.0527 (10)	
H47	0.7416	0.4984	0.4743	0.063*	
C48	0.8395 (7)	0.3934 (3)	0.42804 (18)	0.0557 (11)	
H48	0.9812	0.3948	0.4516	0.067*	
C49	0.7751 (7)	0.3310 (2)	0.38076 (17)	0.0507 (10)	
H49	0.8713	0.2887	0.3717	0.061*	
C50	0.5700 (7)	0.3294 (2)	0.34612 (16)	0.0446 (9)	
H50	0.5262	0.2861	0.3132	0.053*	
C51	0.7877 (6)	0.84135 (17)	0.29690 (18)	0.101 (2)	
H51	0.6861	0.8779	0.3170	0.121*	
C52	0.9878 (6)	0.86602 (14)	0.27527 (19)	0.0911 (18)	
H52	1.0230	0.9195	0.2805	0.109*	
C53	1.1364 (5)	0.8125 (2)	0.24592 (18)	0.097 (2)	
H53	1.2732	0.8293	0.2311	0.117*	
C54	1.0849 (6)	0.73429 (18)	0.23820 (15)	0.0828 (16)	
H54	1.1864	0.6977	0.2181	0.099*	
C55	0.8848 (6)	0.70963 (13)	0.25983 (16)	0.0666 (13)	
H55	0.8495	0.6562	0.2546	0.080*	
C56	0.7362 (5)	0.76315 (19)	0.28918 (17)	0.0800 (16)	
H56	0.5994	0.7463	0.3040	0.096*	
C57	1.2877 (8)	0.0159 (4)	0.4782 (3)	0.0794 (17)	
H57	1.1403	0.0270	0.4629	0.095*	
C58	1.3994 (10)	0.0665 (3)	0.5279 (3)	0.0766 (15)	
H58	1.3291	0.1122	0.5471	0.092*	
C59	1.6137 (11)	0.0509 (4)	0.5499 (3)	0.0865 (17)	
H59	1.6928	0.0860	0.5841	0.104*	
N1	0.2293 (4)	−0.08860 (14)	0.05611 (11)	0.0272 (5)	
N2	2.6094 (4)	0.65448 (13)	0.91102 (10)	0.0233 (5)	
N3	2.8130 (5)	0.70501 (16)	0.78374 (11)	0.0348 (6)	
N4	2.2537 (4)	0.57284 (14)	1.00265 (11)	0.0286 (6)	
O1	0.1208 (4)	−0.10055 (12)	0.00485 (9)	0.0358 (5)	
O2	0.4049 (4)	0.09116 (12)	0.22097 (9)	0.0312 (5)	
O3	0.5864 (4)	−0.00577 (12)	0.24535 (9)	0.0334 (5)	

### Atomic displacement parameters (Å^2^)

**Table:**

	*U*^11^	*U*^22^	*U*^33^	*U*^12^	*U*^13^	*U*^23^
C1	0.0219 (14)	0.0236 (14)	0.0216 (14)	0.0012 (11)	0.0000 (11)	0.0047 (11)
C2	0.0209 (13)	0.0238 (14)	0.0228 (14)	−0.0039 (11)	0.0002 (11)	0.0064 (11)
C3	0.0219 (14)	0.0243 (15)	0.0258 (15)	−0.0022 (11)	−0.0030 (11)	0.0070 (12)
C4	0.0251 (14)	0.0224 (14)	0.0275 (15)	−0.0001 (12)	−0.0033 (12)	0.0041 (12)
C5	0.0238 (15)	0.0324 (17)	0.0300 (17)	−0.0001 (13)	−0.0004 (12)	0.0036 (13)
C6	0.0287 (15)	0.0310 (16)	0.0288 (16)	−0.0024 (13)	−0.0009 (12)	0.0104 (13)
C7	0.0213 (14)	0.0265 (16)	0.0251 (15)	−0.0022 (12)	−0.0008 (11)	0.0087 (12)
C8	0.0319 (16)	0.0278 (16)	0.0333 (17)	0.0026 (13)	0.0014 (13)	0.0040 (13)
C9	0.0303 (16)	0.0278 (16)	0.0416 (19)	−0.0068 (13)	−0.0038 (14)	0.0071 (14)
C10	0.0353 (16)	0.0225 (15)	0.0220 (15)	−0.0013 (13)	−0.0093 (12)	0.0020 (12)
C11	0.0404 (18)	0.0417 (19)	0.0226 (16)	−0.0030 (15)	0.0012 (13)	0.0052 (14)
C12	0.0327 (17)	0.0395 (18)	0.0258 (16)	−0.0100 (14)	0.0016 (13)	0.0032 (14)
C13	0.0272 (15)	0.0255 (15)	0.0236 (15)	0.0014 (12)	−0.0021 (12)	0.0069 (12)
C14	0.0333 (17)	0.0409 (18)	0.0224 (16)	−0.0086 (14)	−0.0001 (13)	0.0070 (13)
C15	0.0324 (17)	0.0391 (18)	0.0276 (17)	−0.0108 (14)	−0.0033 (13)	0.0068 (14)
C16	0.0239 (14)	0.0285 (15)	0.0225 (15)	−0.0017 (12)	−0.0031 (11)	0.0067 (12)
C17	0.0350 (17)	0.045 (2)	0.0253 (16)	−0.0097 (15)	0.0019 (13)	0.0069 (14)
C18	0.0294 (16)	0.0439 (19)	0.0306 (17)	−0.0140 (14)	−0.0002 (13)	0.0059 (14)
C19	0.0288 (15)	0.0285 (16)	0.0254 (15)	−0.0020 (13)	−0.0055 (12)	0.0048 (12)
C20	0.0355 (17)	0.045 (2)	0.0228 (16)	−0.0098 (15)	0.0028 (13)	0.0007 (14)
C21	0.0299 (16)	0.0423 (19)	0.0264 (16)	−0.0095 (14)	0.0020 (13)	0.0027 (14)
C22	0.0293 (16)	0.0295 (16)	0.0286 (16)	−0.0029 (13)	−0.0015 (13)	0.0056 (13)
C23	0.0276 (15)	0.0311 (16)	0.0258 (16)	−0.0013 (13)	−0.0005 (12)	0.0057 (13)
C24	0.0282 (15)	0.0269 (15)	0.0247 (15)	−0.0016 (12)	−0.0040 (12)	0.0054 (12)
C25	0.0316 (16)	0.0307 (16)	0.0231 (15)	−0.0040 (13)	−0.0009 (12)	0.0032 (13)
C26	0.0265 (15)	0.0310 (16)	0.0258 (16)	−0.0039 (12)	−0.0012 (12)	0.0052 (13)
C27	0.0261 (14)	0.0180 (13)	0.0249 (15)	−0.0002 (11)	−0.0022 (11)	0.0047 (11)
C28	0.0247 (14)	0.0276 (15)	0.0236 (15)	−0.0021 (12)	−0.0014 (11)	0.0068 (12)
C29	0.0247 (14)	0.0260 (15)	0.0267 (16)	−0.0027 (12)	−0.0020 (12)	0.0051 (12)
C30	0.0242 (14)	0.0191 (14)	0.0244 (15)	−0.0009 (11)	−0.0001 (11)	0.0046 (11)
C31	0.0284 (15)	0.0235 (14)	0.0223 (15)	−0.0021 (12)	0.0013 (12)	0.0072 (12)
C32	0.0274 (15)	0.0222 (14)	0.0221 (15)	−0.0009 (12)	0.0000 (11)	0.0061 (11)
C33	0.0267 (14)	0.0207 (14)	0.0250 (15)	0.0011 (12)	0.0004 (12)	0.0073 (12)
C34	0.0270 (15)	0.0223 (14)	0.0243 (15)	−0.0023 (12)	0.0030 (12)	0.0075 (12)
C35	0.0292 (15)	0.0200 (14)	0.0238 (15)	−0.0009 (12)	0.0025 (12)	0.0031 (11)
C36	0.0308 (16)	0.0279 (16)	0.0271 (16)	−0.0054 (13)	−0.0001 (12)	0.0061 (13)
C37	0.0298 (16)	0.0253 (15)	0.0340 (17)	−0.0067 (13)	0.0000 (13)	0.0033 (13)
C38	0.0341 (17)	0.0300 (16)	0.0363 (18)	−0.0069 (13)	0.0060 (14)	0.0088 (14)
C39	0.046 (2)	0.044 (2)	0.0284 (17)	−0.0093 (16)	0.0064 (14)	0.0140 (15)
C40	0.0274 (15)	0.0191 (14)	0.0237 (15)	−0.0020 (11)	0.0011 (12)	0.0055 (11)
C41	0.0294 (15)	0.0266 (15)	0.0276 (16)	−0.0025 (12)	−0.0009 (12)	0.0089 (12)
C42	0.0380 (17)	0.0319 (17)	0.0252 (16)	0.0019 (14)	−0.0027 (13)	0.0075 (13)
C43	0.0464 (19)	0.0323 (17)	0.0230 (16)	0.0016 (14)	0.0025 (14)	0.0108 (13)
C44	0.0402 (18)	0.0302 (16)	0.0298 (17)	−0.0001 (14)	0.0037 (14)	0.0135 (13)
C45	0.0380 (19)	0.057 (2)	0.036 (2)	0.0008 (17)	0.0055 (15)	0.0213 (17)
C46	0.061 (2)	0.045 (2)	0.041 (2)	0.0118 (18)	0.0159 (18)	0.0194 (17)
C47	0.063 (3)	0.053 (2)	0.037 (2)	−0.012 (2)	0.0112 (18)	0.0051 (18)
C48	0.037 (2)	0.090 (3)	0.041 (2)	0.004 (2)	0.0034 (17)	0.021 (2)
C49	0.061 (2)	0.060 (3)	0.042 (2)	0.027 (2)	0.0204 (19)	0.026 (2)
C50	0.061 (2)	0.040 (2)	0.0322 (19)	−0.0056 (18)	0.0072 (17)	0.0103 (15)
C51	0.116 (5)	0.067 (3)	0.141 (6)	0.038 (3)	0.062 (4)	0.046 (4)
C52	0.081 (4)	0.060 (3)	0.152 (6)	0.009 (3)	0.020 (4)	0.059 (4)
C53	0.073 (3)	0.113 (5)	0.149 (6)	0.026 (3)	0.046 (4)	0.097 (4)
C54	0.099 (4)	0.069 (3)	0.088 (4)	0.040 (3)	0.028 (3)	0.025 (3)
C55	0.071 (3)	0.046 (2)	0.084 (3)	0.002 (2)	−0.015 (3)	0.025 (2)
C56	0.061 (3)	0.084 (4)	0.121 (5)	0.013 (3)	0.024 (3)	0.068 (3)
C57	0.048 (3)	0.109 (4)	0.119 (5)	0.026 (3)	0.031 (3)	0.088 (4)
C58	0.082 (4)	0.077 (4)	0.095 (4)	0.024 (3)	0.035 (3)	0.057 (3)
C59	0.093 (4)	0.102 (4)	0.088 (4)	−0.014 (4)	0.015 (3)	0.068 (4)
N1	0.0300 (13)	0.0256 (13)	0.0215 (13)	0.0024 (10)	−0.0069 (10)	0.0008 (10)
N2	0.0258 (12)	0.0205 (12)	0.0223 (12)	−0.0009 (10)	0.0013 (10)	0.0043 (10)
N3	0.0401 (15)	0.0379 (15)	0.0256 (14)	−0.0101 (12)	0.0026 (11)	0.0092 (12)
N4	0.0301 (13)	0.0276 (13)	0.0286 (14)	−0.0056 (11)	0.0028 (10)	0.0093 (11)
O1	0.0413 (13)	0.0371 (12)	0.0238 (11)	0.0037 (10)	−0.0113 (9)	0.0023 (9)
O2	0.0334 (11)	0.0276 (12)	0.0290 (12)	0.0003 (9)	−0.0022 (9)	0.0028 (9)
O3	0.0464 (13)	0.0265 (11)	0.0220 (11)	−0.0053 (10)	−0.0143 (9)	0.0030 (9)

### Geometric parameters (Å, º)

**Table:**

C1—C2	1.519 (4)	C30—C34	1.395 (4)
C1—C5	1.526 (4)	C31—H31	0.9500
C1—C6	1.522 (4)	C31—C32	1.401 (4)
C1—N1	1.489 (4)	C32—C35	1.483 (4)
C2—C3	1.329 (4)	C32—N2	1.341 (4)
C2—C7	1.473 (4)	C33—C34	1.389 (4)
C3—H3	0.9500	C33—C40	1.496 (4)
C3—C4	1.498 (4)	C33—N2	1.346 (4)
C4—C8	1.526 (4)	C34—H34	0.9500
C4—C9	1.535 (4)	C35—C36	1.384 (4)
C4—N1	1.479 (4)	C35—N3	1.342 (4)
C5—H5A	0.9800	C36—H36	0.9500
C5—H5B	0.9800	C36—C37	1.389 (4)
C5—H5C	0.9800	C37—H37	0.9500
C6—H6A	0.9800	C37—C38	1.370 (4)
C6—H6B	0.9800	C38—H38	0.9500
C6—H6C	0.9800	C38—C39	1.382 (5)
C7—O2	1.203 (3)	C39—H39	0.9500
C7—O3	1.361 (3)	C39—N3	1.338 (4)
C8—H8A	0.9800	C40—C41	1.397 (4)
C8—H8B	0.9800	C40—N4	1.346 (4)
C8—H8C	0.9800	C41—H41	0.9500
C9—H9A	0.9800	C41—C42	1.385 (4)
C9—H9B	0.9800	C42—H42	0.9500
C9—H9C	0.9800	C42—C43	1.383 (4)
C10—C11	1.375 (4)	C43—H43	0.9500
C10—C15	1.367 (4)	C43—C44	1.382 (4)
C10—O3	1.413 (3)	C44—H44	0.9500
C11—H11	0.9500	C44—N4	1.339 (4)
C11—C12	1.381 (4)	C45—H45	0.9500
C12—H12	0.9500	C45—C46	1.371 (5)
C12—C13	1.394 (4)	C45—C50	1.370 (5)
C13—C14	1.399 (4)	C46—H46	0.9500
C13—C16	1.487 (4)	C46—C47	1.381 (6)
C14—H14	0.9500	C47—H47	0.9500
C14—C15	1.391 (4)	C47—C48	1.387 (6)
C15—H15	0.9500	C48—H48	0.9500
C16—C17	1.398 (4)	C48—C49	1.367 (6)
C16—C21	1.393 (4)	C49—H49	0.9500
C17—H17	0.9500	C49—C50	1.382 (5)
C17—C18	1.365 (4)	C50—H50	0.9500
C18—H18	0.9500	C51—H51	0.9500
C18—C19	1.396 (4)	C51—C52	1.3900
C19—C20	1.397 (4)	C51—C56	1.3900
C19—C22	1.437 (4)	C52—H52	0.9500
C20—H20	0.9500	C52—C53	1.3900
C20—C21	1.388 (4)	C53—H53	0.9500
C21—H21	0.9500	C53—C54	1.3900
C22—C23	1.195 (4)	C54—H54	0.9500
C23—C24	1.435 (4)	C54—C55	1.3900
C24—C25	1.405 (4)	C55—H55	0.9500
C24—C29	1.398 (4)	C55—C56	1.3900
C25—H25	0.9500	C56—H56	0.9500
C25—C26	1.384 (4)	C57—H57	0.9500
C26—H26	0.9500	C57—C58	1.370 (8)
C26—C27	1.399 (4)	C57—C59^i^	1.376 (8)
C27—C28	1.402 (4)	C58—H58	0.9500
C27—C30	1.489 (4)	C58—C59	1.376 (7)
C28—H28	0.9500	C59—C57^i^	1.376 (8)
C28—C29	1.383 (4)	C59—H59	0.9500
C29—H29	0.9500	N1—O1	1.274 (3)
C30—C31	1.398 (4)	O1—O1^ii^	4.004 (4)
			
C2—C1—C5	113.1 (2)	C28—C29—C24	120.7 (3)
C2—C1—C6	114.8 (2)	C28—C29—H29	119.6
C6—C1—C5	110.6 (2)	C31—C30—C27	121.5 (3)
N1—C1—C2	98.8 (2)	C34—C30—C27	121.0 (2)
N1—C1—C5	108.9 (2)	C34—C30—C31	117.4 (2)
N1—C1—C6	109.8 (2)	C30—C31—H31	120.4
C3—C2—C1	112.7 (2)	C30—C31—C32	119.2 (3)
C3—C2—C7	125.8 (3)	C32—C31—H31	120.4
C7—C2—C1	121.4 (2)	C31—C32—C35	120.5 (3)
C2—C3—H3	123.3	N2—C32—C31	123.3 (3)
C2—C3—C4	113.4 (3)	N2—C32—C35	116.2 (2)
C4—C3—H3	123.3	C34—C33—C40	120.2 (2)
C3—C4—C8	113.1 (2)	N2—C33—C34	123.6 (3)
C3—C4—C9	112.5 (2)	N2—C33—C40	116.2 (2)
C8—C4—C9	110.9 (2)	C30—C34—H34	120.3
N1—C4—C3	99.6 (2)	C33—C34—C30	119.4 (3)
N1—C4—C8	110.1 (2)	C33—C34—H34	120.3
N1—C4—C9	110.2 (2)	C36—C35—C32	121.6 (3)
C1—C5—H5A	109.5	N3—C35—C32	116.1 (2)
C1—C5—H5B	109.5	N3—C35—C36	122.3 (3)
C1—C5—H5C	109.5	C35—C36—H36	120.3
H5A—C5—H5B	109.5	C35—C36—C37	119.4 (3)
H5A—C5—H5C	109.5	C37—C36—H36	120.3
H5B—C5—H5C	109.5	C36—C37—H37	120.6
C1—C6—H6A	109.5	C38—C37—C36	118.9 (3)
C1—C6—H6B	109.5	C38—C37—H37	120.6
C1—C6—H6C	109.5	C37—C38—H38	120.9
H6A—C6—H6B	109.5	C37—C38—C39	118.1 (3)
H6A—C6—H6C	109.5	C39—C38—H38	120.9
H6B—C6—H6C	109.5	C38—C39—H39	117.9
O2—C7—C2	125.5 (3)	N3—C39—C38	124.2 (3)
O2—C7—O3	123.5 (3)	N3—C39—H39	117.9
O3—C7—C2	111.0 (2)	C41—C40—C33	121.0 (3)
C4—C8—H8A	109.5	N4—C40—C33	116.1 (2)
C4—C8—H8B	109.5	N4—C40—C41	122.9 (3)
C4—C8—H8C	109.5	C40—C41—H41	120.7
H8A—C8—H8B	109.5	C42—C41—C40	118.6 (3)
H8A—C8—H8C	109.5	C42—C41—H41	120.7
H8B—C8—H8C	109.5	C41—C42—H42	120.6
C4—C9—H9A	109.5	C43—C42—C41	118.8 (3)
C4—C9—H9B	109.5	C43—C42—H42	120.6
C4—C9—H9C	109.5	C42—C43—H43	120.7
H9A—C9—H9B	109.5	C44—C43—C42	118.7 (3)
H9A—C9—H9C	109.5	C44—C43—H43	120.7
H9B—C9—H9C	109.5	C43—C44—H44	118.0
C11—C10—O3	118.5 (3)	N4—C44—C43	123.9 (3)
C15—C10—C11	121.2 (3)	N4—C44—H44	118.0
C15—C10—O3	120.1 (3)	C46—C45—H45	120.2
C10—C11—H11	120.4	C50—C45—H45	120.2
C10—C11—C12	119.2 (3)	C50—C45—C46	119.7 (4)
C12—C11—H11	120.4	C45—C46—H46	119.7
C11—C12—H12	119.0	C45—C46—C47	120.5 (4)
C11—C12—C13	121.9 (3)	C47—C46—H46	119.7
C13—C12—H12	119.0	C46—C47—H47	120.3
C12—C13—C14	117.0 (3)	C46—C47—C48	119.5 (4)
C12—C13—C16	121.4 (3)	C48—C47—H47	120.3
C14—C13—C16	121.7 (3)	C47—C48—H48	120.1
C13—C14—H14	119.3	C49—C48—C47	119.9 (4)
C15—C14—C13	121.4 (3)	C49—C48—H48	120.1
C15—C14—H14	119.3	C48—C49—H49	120.0
C10—C15—C14	119.3 (3)	C48—C49—C50	120.0 (4)
C10—C15—H15	120.4	C50—C49—H49	120.0
C14—C15—H15	120.4	C45—C50—C49	120.4 (4)
C17—C16—C13	120.9 (3)	C45—C50—H50	119.8
C21—C16—C13	122.5 (3)	C49—C50—H50	119.8
C21—C16—C17	116.6 (3)	C52—C51—H51	120.0
C16—C17—H17	118.8	C52—C51—C56	120.0
C18—C17—C16	122.4 (3)	C56—C51—H51	120.0
C18—C17—H17	118.8	C51—C52—H52	120.0
C17—C18—H18	119.6	C51—C52—C53	120.0
C17—C18—C19	120.8 (3)	C53—C52—H52	120.0
C19—C18—H18	119.6	C52—C53—H53	120.0
C18—C19—C20	118.0 (3)	C54—C53—C52	120.0
C18—C19—C22	119.1 (3)	C54—C53—H53	120.0
C20—C19—C22	122.9 (3)	C53—C54—H54	120.0
C19—C20—H20	119.8	C55—C54—C53	120.0
C21—C20—C19	120.4 (3)	C55—C54—H54	120.0
C21—C20—H20	119.8	C54—C55—H55	120.0
C16—C21—H21	119.1	C54—C55—C56	120.0
C20—C21—C16	121.8 (3)	C56—C55—H55	120.0
C20—C21—H21	119.1	C51—C56—H56	120.0
C23—C22—C19	174.6 (3)	C55—C56—C51	120.0
C22—C23—C24	177.8 (3)	C55—C56—H56	120.0
C25—C24—C23	120.0 (3)	C58—C57—H57	119.6
C29—C24—C23	121.5 (3)	C58—C57—C59^i^	120.8 (5)
C29—C24—C25	118.5 (3)	C59^i^—C57—H57	119.6
C24—C25—H25	119.8	C57—C58—H58	120.1
C26—C25—C24	120.4 (3)	C57—C58—C59	119.8 (5)
C26—C25—H25	119.8	C59—C58—H58	120.1
C25—C26—H26	119.4	C57^i^—C59—H59	120.3
C25—C26—C27	121.2 (3)	C58—C59—C57^i^	119.4 (6)
C27—C26—H26	119.4	C58—C59—H59	120.3
C26—C27—C28	118.1 (3)	C4—N1—C1	115.5 (2)
C26—C27—C30	121.1 (3)	O1—N1—C1	122.4 (2)
C28—C27—C30	120.8 (3)	O1—N1—C4	122.1 (2)
C27—C28—H28	119.5	C32—N2—C33	117.1 (2)
C29—C28—C27	121.0 (3)	C39—N3—C35	117.2 (3)
C29—C28—H28	119.5	C44—N4—C40	117.0 (3)
C24—C29—H29	119.6	C7—O3—C10	116.5 (2)
			
C1—C2—C3—C4	−0.1 (3)	C27—C30—C31—C32	−175.1 (3)
C1—C2—C7—O2	−1.5 (4)	C27—C30—C34—C33	176.3 (3)
C1—C2—C7—O3	179.4 (2)	C28—C27—C30—C31	−158.0 (3)
C2—C1—N1—C4	−0.5 (3)	C28—C27—C30—C34	24.6 (4)
C2—C1—N1—O1	−179.4 (2)	C29—C24—C25—C26	0.2 (5)
C2—C3—C4—C8	116.6 (3)	C30—C27—C28—C29	−177.8 (3)
C2—C3—C4—C9	−116.8 (3)	C30—C31—C32—C35	177.0 (3)
C2—C3—C4—N1	−0.2 (3)	C30—C31—C32—N2	−2.0 (4)
C2—C7—O3—C10	168.2 (2)	C31—C30—C34—C33	−1.2 (4)
C3—C2—C7—O2	176.4 (3)	C31—C32—C35—C36	174.6 (3)
C3—C2—C7—O3	−2.6 (4)	C31—C32—C35—N3	−7.8 (4)
C3—C4—N1—C1	0.4 (3)	C31—C32—N2—C33	0.2 (4)
C3—C4—N1—O1	179.4 (2)	C32—C35—C36—C37	176.1 (3)
C5—C1—C2—C3	115.3 (3)	C32—C35—N3—C39	−175.8 (3)
C5—C1—C2—C7	−66.5 (3)	C33—C40—C41—C42	177.4 (3)
C5—C1—N1—C4	−118.7 (3)	C33—C40—N4—C44	−178.8 (3)
C5—C1—N1—O1	62.4 (3)	C34—C30—C31—C32	2.4 (4)
C6—C1—C2—C3	−116.4 (3)	C34—C33—C40—C41	164.3 (3)
C6—C1—C2—C7	61.8 (3)	C34—C33—C40—N4	−15.8 (4)
C6—C1—N1—C4	120.0 (3)	C34—C33—N2—C32	1.1 (4)
C6—C1—N1—O1	−58.9 (3)	C35—C32—N2—C33	−178.8 (2)
C7—C2—C3—C4	−178.2 (3)	C35—C36—C37—C38	−0.1 (5)
C8—C4—N1—C1	−118.6 (3)	C36—C35—N3—C39	1.8 (5)
C8—C4—N1—O1	60.4 (3)	C36—C37—C38—C39	1.2 (5)
C9—C4—N1—C1	118.8 (3)	C37—C38—C39—N3	−0.8 (5)
C9—C4—N1—O1	−62.3 (4)	C38—C39—N3—C35	−0.7 (5)
C10—C11—C12—C13	−0.6 (5)	C40—C33—C34—C30	−178.3 (3)
C11—C10—C15—C14	0.5 (5)	C40—C33—N2—C32	179.0 (2)
C11—C10—O3—C7	−80.9 (4)	C40—C41—C42—C43	2.1 (4)
C11—C12—C13—C14	1.5 (5)	C41—C40—N4—C44	1.1 (4)
C11—C12—C13—C16	−178.0 (3)	C41—C42—C43—C44	−0.4 (5)
C12—C13—C14—C15	−1.5 (5)	C42—C43—C44—N4	−1.1 (5)
C12—C13—C16—C17	−4.5 (5)	C43—C44—N4—C40	0.8 (5)
C12—C13—C16—C21	176.1 (3)	C45—C46—C47—C48	−0.9 (6)
C13—C14—C15—C10	0.5 (5)	C46—C45—C50—C49	−0.6 (5)
C13—C16—C17—C18	−178.4 (3)	C46—C47—C48—C49	0.0 (6)
C13—C16—C21—C20	178.4 (3)	C47—C48—C49—C50	0.6 (6)
C14—C13—C16—C17	176.0 (3)	C48—C49—C50—C45	−0.3 (5)
C14—C13—C16—C21	−3.4 (5)	C50—C45—C46—C47	1.2 (5)
C15—C10—C11—C12	−0.5 (5)	C51—C52—C53—C54	0.0
C15—C10—O3—C7	103.6 (3)	C52—C51—C56—C55	0.0
C16—C13—C14—C15	178.0 (3)	C52—C53—C54—C55	0.0
C16—C17—C18—C19	0.4 (5)	C53—C54—C55—C56	0.0
C17—C16—C21—C20	−1.0 (5)	C54—C55—C56—C51	0.0
C17—C18—C19—C20	−1.8 (5)	C56—C51—C52—C53	0.0
C17—C18—C19—C22	176.8 (3)	C57—C58—C59—C57^i^	−0.5 (8)
C18—C19—C20—C21	1.8 (5)	C59^i^—C57—C58—C59	0.5 (8)
C19—C20—C21—C16	−0.4 (5)	N1—C1—C2—C3	0.4 (3)
C21—C16—C17—C18	1.0 (5)	N1—C1—C2—C7	178.5 (2)
C22—C19—C20—C21	−176.7 (3)	N2—C32—C35—C36	−6.4 (4)
C23—C24—C25—C26	−179.5 (3)	N2—C32—C35—N3	171.3 (3)
C23—C24—C29—C28	178.9 (3)	N2—C33—C34—C30	−0.5 (4)
C24—C25—C26—C27	1.0 (5)	N2—C33—C40—C41	−13.7 (4)
C25—C24—C29—C28	−0.8 (4)	N2—C33—C40—N4	166.2 (2)
C25—C26—C27—C28	−1.5 (4)	N3—C35—C36—C37	−1.5 (5)
C25—C26—C27—C30	177.1 (3)	N4—C40—C41—C42	−2.5 (4)
C26—C27—C28—C29	0.9 (4)	O2—C7—O3—C10	−10.8 (4)
C26—C27—C30—C31	23.4 (4)	O3—C10—C11—C12	−175.9 (3)
C26—C27—C30—C34	−154.0 (3)	O3—C10—C15—C14	175.9 (3)
C27—C28—C29—C24	0.3 (4)		

Symmetry codes: (i) −*x*+3, −*y*, −*z*+1; (ii) −*x*, −*y*, −*z*.

### Hydrogen-bond geometry (Å, º)

Cg4, Cg7 and Cg10 are the centroids of pyridine ring N4/C40–C44, spacer ring C24–C29 and benzene ring C54–C59, respectively.

**Table:**

*D*—H···*A*	*D*—H	H···*A*	*D*···*A*	*D*—H···*A*
C37—H37···O1^iii^	0.95	2.65	3.228 (4)	120
C38—H38···O2^iv^	0.95	2.55	3.485 (4)	169
C6—H6*C*···O1^ii^	0.98	2.61	3.499 (4)	151
C9—H9*B*···*Cg*4^v^	0.96	2.79	3.602 (4)	140
C14—H14···*Cg*10^vi^	0.95	2.88	3.608 (4)	134
C14—H14···*Cg*10^vii^	0.95	2.88	3.608 (4)	134
C55—H55···*Cg*7^viii^	0.95	2.90	3.680 (3)	140

Symmetry codes: (ii) −*x*, −*y*, −*z*; (iii) *x*+3, *y*+1, *z*+1; (iv) −*x*+4, −*y*+1, −*z*+1; (v) *x*−2, *y*−1, *z*−1; (vi) *x*−1, *y*, *z*; (vii) −*x*+2, −*y*, −*z*+1; (viii) −*x*+3, −*y*+1, −*z*+1.

## References

[bb1] Ackermann, K., Giannoulis, A., Cordes, D. B., Slawin, A. M. Z. & Bode, B. E. (2015). *Chem. Commun.* **51**, 5257–5260. 10.1039/c4cc08656b 25587579

[bb2] Bessel, C. A., See, R. F., Jameson, D. L., Churchill, M. R. & Takeuchi, K. J. (1992). *J. Chem. Soc. Dalton Trans.* pp. 3223–3228.

[bb3] Blessing, R. H. (1995). *Acta Cryst.* A**51**, 33–38. 10.1107/s0108767394005726 7702794

[bb4] Bode, E. B., Plackmeyer, J., Prisner, T. F. & Schiemann, O. (2008). *J. Phys. Chem. A*, **112**, 5064–5073. 10.1021/jp710504k 18491846

[bb5] Constable, E. C., Baum, G., Bill, E., Dyson, R., van Eldik, R., Fenske, D., Kaderli, D., Morris, D., Neubrand, A., Neuburger, M., Smith, D. R., Wieghardt, K., Zehnder, M. & Zuberbühler, A. D. (1999). *Chem. Eur. J.* **5**, 498–508.

[bb6] Dolomanov, O. V., Bourhis, L. J., Gildea, R. J., Howard, J. A. K. & Puschmann, H. (2009). *J. Appl. Cryst.* **42**, 339–341.

[bb7] Eryazici, I., Moorefield, C. N., Durmus, S. & Newkome, G. R. (2006). *J. Org. Chem.* **71**, 1009–1014. 10.1021/jo052036l 16438513

[bb8] Fallahpour, R.-A., Neuburger, M. & Zehnder, M. (1999). *Polyhedron*, **18**, 2445–2454.

[bb9] Fey, T., Fischer, H., Bachmann, S., Albert, K. & Bolm, C. (2001). *J. Org. Chem.* **66**, 8154–8159. 10.1021/jo010535q 11722219

[bb10] Fink, D. W. & Ohnesorge, W. E. (1970). *J. Phys. Chem.* **74**, 72–77.

[bb11] Folgado, J. V., Henke, W., Allmann, R., Stratemeier, H., Beltrán-Porter, D., Rojo, T. & Reinen, D. (1990). *Inorg. Chem.* **29**, 2035–2042.

[bb12] Fritscher, J., Beyer, M. & Schiemann, O. (2002). *Chem. Phys. Lett.* **364**, 393–401.

[bb13] Grave, C., Lentz, D., Schäfer, A., Samorì, P., Rabe, P. J., Franke, P. & Schlüter, A. D. (2003). *J. Am. Chem. Soc.* **125**, 6907–6918. 10.1021/ja034029p 12783543

[bb14] Groom, C. R. & Allen, F. H. (2014). *Angew. Chem. Int. Ed.* **53**, 662–671. 10.1002/anie.201306438 24382699

[bb15] Grosshenny, V. & Ziessel, R. (1993). *J. Organomet. Chem.* **453**, C19–C22.

[bb16] Hogg, R. & Wilkins, R. G. (1962). *J. Chem. Soc.* pp. 341–350.

[bb17] Hoover, J. M. & Stahl, S. S. (2011). *J. Am. Chem. Soc.* **133**, 16901–16910. 10.1021/ja206230h PMC3197761 21861488

[bb18] Janiak, C. (2000). *J. Chem. Soc. Dalton Trans.* pp. 3885–3896.

[bb19] Macrae, C. F., Bruno, I. J., Chisholm, J. A., Edgington, P. R., McCabe, P., Pidcock, E., Rodriguez-Monge, L., Taylor, R., van de Streek, J. & Wood, P. A. (2008). *J. Appl. Cryst.* **41**, 466–470.

[bb20] Margraf, D., Schuetz, D., Prisner, T. F. & Bats, J. W. (2009). *Acta Cryst.* E**65**, o1784. 10.1107/S1600536809024659 PMC2977196 21583490

[bb21] Meyer, A., Schnakenburg, G., Glaum, R. & Schiemann, O. (2015*b*). *Inorg. Chem.* **54**, 8456–8464. 10.1021/acs.inorgchem.5b01157 26275138

[bb22] Meyer, A., Wiecek, J., Schnakenburg, G. & Schiemann, O. (2015*a*). *Acta Cryst.* E**71**, 870–874. 10.1107/S2056989015012086 PMC4518943 26279889

[bb23] Murguly, E., Norsten, T. B. & Branda, N. (1999). *J. Chem. Soc. Perkin Trans. 2*, pp. 2789–2794.

[bb24] Nakamoto, K. (1960). *J. Phys. Chem.* **64**, 1420–1425.

[bb25] Narr, E., Godt, A. & Jeschke, G. (2002). *Angew. Chem. Int. Ed.* **41**, 3907–3910. 10.1002/1521-3773(20021018)41:20<3907::AID-ANIE3907>3.0.CO;2-T 12386888

[bb26] Otwinowski, Z. & Minor, W. (1997). *Methods in Enzymology*, Vol. 276, *Macromolecular Crystallography*, Part A, edited by C. W. Carter Jr & R. M. Sweet, pp. 307–326. New York: Academic Press.

[bb27] Rajca, A., Mukherjee, S., Pink, M. & Rajca, S. (2006). *J. Am. Chem. Soc.* **128**, 13497–13507. 10.1021/ja063567+ PMC2561304 17031963

[bb28] Reginsson, G. W. & Schiemann, O. (2011). *Biochem. Soc. Trans.* **39**, 128–139. 10.1042/BST0390128 21265760

[bb29] Schuetz, D., Margraf, D., Prisner, T. F. & Bats, J. W. (2010). *Acta Cryst.* E**66**, o729–o730. 10.1107/S1600536810007294 PMC2983865 21580576

[bb30] Sheldrick, G. M. (2008). *Acta Cryst.* A**64**, 112–122. 10.1107/S0108767307043930 18156677

[bb31] Sheldrick, G. M. (2015). *Acta Cryst.* C**71**, 3–8.

